# Do clock-in for physical exercise in campus help in building undergraduate exercise habits? Evidence from undergraduates in Henan, China

**DOI:** 10.3389/fpubh.2026.1844179

**Published:** 2026-06-01

**Authors:** Duxuan Zhu, Xiao Ma, Huiying Fan, Qiang Yuan

**Affiliations:** 1 School of Physical Education, Shanghai University of Sport, Shanghai, China; 2 School of Physical Education, Shanghai Normal University, Shanghai, China; 3 School of Physical Education, Zhoukou Normal University, Zhoukou, China

**Keywords:** exercise habits, mediation analysis, moderate-to-vigorous physical activity, physical exercise clock-in, undergraduates

## Abstract

**Background:**

Physical inactivity among undergraduates has raised increasing public health concern. Many Chinese universities have implemented physical exercise clock-in programs to promote regular participation. This study examined whether participation frequency is associated with higher moderate-to-vigorous physical activity (MVPA) and whether exercise habits mediate this relationship.

**Methods:**

A cross-sectional survey was conducted between April and May 2025 among 468 s-year undergraduates from a comprehensive university in Henan Province, China. MVPA was assessed based on self-reported frequency and duration of MVPA during the past seven days. Participation in the clock-in program was categorized into non-participation, occasional participation, and nearly/full attendance. Multiple linear regression models were used to examine associations. Propensity score matching (nearest neighbor, radius, and kernel matching) was applied to test robustness. Mediation analysis was performed via nonparametric percentile bootstrapping (5,000 resamples) to examine the indirect effect of exercise habits.

**Results:**

High-frequency participation in the clock-in program was significantly associated with higher MVPA levels (*b* = 18.751, 95% CI [7.08, 30.42], *p* < 0.05), whereas occasional participation showed no significant effect. Propensity score matching yielded consistent results, with average treatment effects ranging from 16.34 to 19.48 min. Exercise habits partially mediated the relationship between high-frequency participation and MVPA (indirect effect = 5.99, 95% CI [2.50, 10.40]). Additionally, gender-stratified subgroup analysis revealed a consistent pattern of this mediating effect across male and female undergraduates.

**Conclusion:**

High-frequency participation in the physical exercise clock-in program is positively associated with greater physical activity among undergraduates, partly through exercise habits. Structured institutional physical activity programs may be correlated with more sustainable exercise behaviors in undergraduate populations.

## Introduction

Physical activity plays a crucial role in promoting physical and mental health across all age groups. While most prior studies have focused on children and older adults—populations that require positive physical experiences to develop healthy habits or maintain successful aging—individuals in their twenties are often considered to be at the peak of their physical health, with greater freedom and leisure time to engage in exercise (1, 2). They are also presumed to possess substantial knowledge about the benefits of physical activity for well-being and happiness (3). As such, one would expect this group to participate regularly in physical exercise for both enjoyment and health maintenance. However, empirical evidence reveals a global decline in physical activity levels among young adults, accompanied by a marked decrease in physical fitness. The *Annual Report on Development of Youth Sports in China (2016)* demonstrated that Chinese undergraduates exhibit significantly lower levels of physical fitness, particularly endurance and strength, than primary and secondary school students do (4). Evidence from a cohort of 58,472 undergraduates in Anhui, China, revealed that the rate of their body weight increased, surpassing that of their height, which led to a notable increase in the proportion of overweight or obese undergraduates (5).

To address this growing public health concern, universities in China have introduced a mandatory daily physical exercise “clock-in” program. Drawing on successful strategies used in elementary schools—where increased opportunities for physical engagement support habit formation—this initiative leverages the institutional setting for scalable implementation. The clock-in system functions as a supplement to the standard physical education curriculum, aiming to encourage consistent participation rather than relying solely on personal motivation. Undergraduates are required by the university to complete a set amount of daily physical activity at designated campus facilities, with attendance and duration recorded via campus ID cards or smartphone apps to accumulate credits. While some institutions have implemented the program similarly to traditional sports clubs, without mandating specific activities, others have adopted more structured approaches. In 2017, Nankai University, a leading institution in China, launched an official clock-in activity. Performance is tracked through a mobile application and contributes 10% to undergraduates’ overall physical education grade. That same year, Beijing Normal University launched a comparable initiative. The following year, Zhejiang University introduced its own enhanced version, incorporating total running distance into 15% of the final physical education score. Distance accumulation is monitored via a smartphone app, with male undergraduates required to complete 168 km and female undergraduates 120 km per semester.

However, the primary objective of the clock-in program extends beyond the increasing undergraduates’ physical activity levels, to actively shape sustainable exercise behaviors. From a behavioral perspective, habit formation critically depends on the repeated pairing of environmental cues with consistent behavioral responses and their associated outcomes (6). In the initial stages, external enforcement serves a facilitative, scaffolding function—helping bridge the gap between intention and action (7, 8). Empirical evidence indicates that approximately 66 days of continuous, contextually stable repetition are sufficient to establish a robust exercise habit (9). Once internalized and automated, such behavior exerts a substantially stronger influence on overall physical activity levels and manifests in higher execution frequency (10). Consequently, when undergraduates repeatedly perform a specific movement pattern under consistent temporal and spatial conditions, the behavior gradually transitions from conscious, effortful regulation to an automatic, cue-triggered response—culminating a stable exercise habit. Students with established exercise habits report significantly greater total weekly exercise time than their peers without such habits and demonstrate greater resilience in maintaining physical activity levels—even under high academic pressure (11). Moreover, the habit-building process not only stabilizes behavioral enactment but also enriches subjective experience (e.g., enjoyment, competence perception), thereby indirectly strengthening exercise self-efficacy and willingness to engage (12). This dynamic interplay fosters a positive feedback loop that supports long-term, sustainable exercise engagement. Collectively, these theoretical foundations and empirical findings offer some support for the notion that clock-in programs may help promote exercise habit formation among undergraduates—particularly through structured behavioral repetition and the reinforcement of contextual cues.

The literature has focused predominantly on quantifying undergraduates’ physical activity accumulation—across population coverage, session duration, and exercise content (13–16)—yet has largely overlooked whether such programs meaningfully contribute to habit formation itself. Critics contend that students primarily comply to fulfill credit requirements or meet performance evaluations—not out of intrinsic interest in health or enjoyment. Such extrinsic pressure risks undermining intrinsic motivation and impeding the internalization of physical activity motives (12, 17). This concern is empirically corroborated by a longitudinal study showing that undergraduates mandated to participate in community service during university reported significantly lower subsequent volunteering intentions than those who volunteered voluntarily—particularly among those initially reluctant to engage (18). These findings suggest that coercion may attenuate behavioral intentions and diminish long-term engagement efficacy. Conversely, proponents argue that, given many undergraduates approach physical activity as a task-oriented, instrumental activity rather than a self-determined pursuit, compulsory participation—when thoughtfully designed—can serve as a necessary catalyst (7, 8). Without such a structure, undergraduates may neither initiate nor persist in exercise, thereby missing opportunities to develop the foundational skills, knowledge, and positive affective associations essential for eventual autonomous engagement.

There is no doubt that the clock-in program positively influences undergraduates’ overall physical activity; however, the relationship between the clock-in program and their self-initiated exercise behaviors remains to be discussed. Evidence from universities and institutions implementing such programs suggests that undergraduates’ total physical activity comprises two conceptually distinct components: structured participation in the clock-in program and autonomous, self-initiated regular exercise. While greater clock-in engagement consistently correlates with higher aggregate physical activity levels, it remains empirically unresolved whether—and to what extent—this mandatory intervention meaningfully shapes habitual, volitional exercise behavior. A statistically significant positive association would indicate that external regulation does not inherently impede long-term behavioral persistence (8); In the domain of physical activity, it may facilitate gradual internalization through repeated practice, thereby promoting the emergence of self-sustained exercise patterns (19). In contrast, a nonsignificant or negative association would imply that clock-in mandates exert minimal influence on intrinsic exercise habits—and may even undermine undergraduates’ autonomous motivation or attitudes toward physical activity. Such evidence would call for a critical reassessment of the program’s theoretical foundations, operational design, and institutional implementation. Accordingly, this study addresses two interrelated research objectives: (1) to explore the relationship between physical exercise clock-in programs and undergraduates’ MVPA levels; and (2) to examine whether exercise habits mediate the relationship between clock-in participation and physical activity level—thereby generating empirically grounded insights to inform the evidence-based design and context-sensitive implementation of such programs in higher education settings.

## Materials and methods

### Date source

The data for this study were collected from undergraduates enrolled at a comprehensive university in Henan Province, China. The university has established a relatively well-developed physical exercise clock-in program, requiring undergraduates to complete a prescribed number of exercise clock-in each semester. The completion of these clock-ins is included in the evaluation of physical education course grades, with campus running being the main form of implementation.

The questionnaire survey was conducted between April and May 2025, using an electronic format distributed and collected through a unified online platform. The participants were second-year undergraduates who had already completed one full academic year of campus running clock-in. Participation in the survey was voluntary and anonymous, and informed consent was obtained from all participants prior to data collection. The questionnaire items used in this study are available in the Supplementary material.

A total of 504 questionnaires were returned. During data cleaning, the following exclusion criteria were applied: (1) questionnaires with evident patterned or careless responses and (2) questionnaires containing logically inconsistent answers. After screening, 468 valid questionnaires were retained (111 males and 357 females), yielding an effective response rate of 92.86%.

### Dependent variable

The dependent variable in this study was MVPA, which reflects the overall level of physical activity among undergraduates. Compared with light physical activity, MVPA provides more explicit and stable health benefits in improving cardiorespiratory function, promoting physical fitness, and preventing chronic diseases (1, 20, 21). It is also the most commonly used core indicator in physical activity surveillance and intervention research worldwide.

The measurement items were adapted from the International Physical Activity Questionnaire (IPAQ)-Short Form, which has demonstrated acceptable reliability and validity in adult populations (22). The adapted scale used two items to assess MVPA: (1) *During the past seven days, on how many days and for approximately how long each day did you engage in vigorous physical activity* (e.g.*, running, heavy weight lifting*)? (2) *During the past seven days, on how many days and for approximately how long each day did you engage in moderate physical activity* (e.g.*, biking, brisk walking*)? The participants reported the number of days and the average duration of their participation in both types of physical activity during the previous seven days. During data processing, the total minutes spent on moderate and vigorous physical activity over the seven days were summed and then divided by seven to calculate the mean daily minutes of MVPA for each participant.

### Independent variables

The independent variable in this study was physical exercise clock-in. The self-reported item measuring physical exercise clock-in was: *How often have you participated in the university’s exercise clock-in program this semester?* The response options were *as follows*: *Never participated*, *occasionally participated*, and *almost full attendance or full attendance*.

### Other variables

Exercise habit was a key variable in the influence pathway and was represented by the frequency of autonomous physical exercise. Autonomous physical exercise excluded regular physical education classes and on-campus exercise clock-in. The self-reported item measuring this variable was: *Apart from physical education classes and on-campus exercise clock-in, how many times per week do you engage in physical exercise?* The response options were *as follows*: *0*, *1*, *2*, *3*, *4*, *5*, *6*, *or 7 times*.

In addition, the active sporting population was included as a control variable, as it is also a principal factor influencing the average daily duration of MVPA. The criterion for defining the active sporting population was participation in MVPA at least three times per week, with each session lasting at least 30 min, and excluding physical education classes and on-campus exercise clock-in. Furthermore, individuals’ perceptions of the natural and social environment, as well as their behavioral patterns, may differ by gender. Moreover, significant disparities in behavioral health outcomes have been observed between urban and rural populations, which has been recognized as an important public health concern. Therefore, gender and residential location were included as control variables in this study (23, 24). Descriptions of the main variables are presented in Table 1, and their descriptive statistics are shown in Table 2.

**Table 1:** Description of the main variables.

Variable	Symbol	Description
Moderate-to-vigorous physical activity	*mvpa*	Average daily duration of moderate-to-vigorous physical activity (minutes)
Physical exercise clock-in	*attendance*	Participation in exercise clock-in during the semester: 0 = never participated, 1 = occasionally participated, 2 = almost full attendance or full attendance
Exercise habit	*selfexercise*	Number of times participating in autonomous physical exercise per week
Active sporting population	*sportsactive*	1 = meets all of the following criteria: autonomous exercise ≥ 3 times/week, duration ≥ 30 min/session, intensity ≥ moderate; 0 = otherwise
Gender	*gender*	0 = female, 1 = male
Residential location	*residence*	0 = urban, 1 = rural

**Table 2:** Descriptive statistics of the main variables.

Variable	Samples	Mean	SD	Max	Min
*mvpa*	468	57.15	49.387	291.43	0
*attendance*	468	1.35	0.764	2	0
*selfexercise*	468	2.24	2.255	7	0
*sportactive*	468	0.15	0.355	1	0
*gender*	468	0.24	0.426	1	0
*residence*	468	0.64	0.488	1	0

### Statistical analysis

All statistical analyses were performed via SPSS software (Version 27.0; SPSS Inc., Chicago, IL, USA) and Stata software (Version 17.0; StataCorp LLC, College Station, TX, USA). (1) Descriptive statistics, including means and standard deviations, were used to summarize the basic characteristics of the participants. (2) Multiple linear regression analyses were performed to examine the relationships between physical exercise clock-in and MVPA, with gender, residential location, and active sporting population included as control variables. (3) To further explore the relationships among physical exercise clock-in, exercise habits, and undergraduates’ MVPA, nonparametric percentile bootstrapping (5,000 resamples) was conducted via the SPSS macro PROCESS (version 2.16) to examine the potential indirect association of exercise habits in the link between physical exercise clock-in and MVPA. (4) The independent variable, physical exercise clock-in, was a categorical variable, with different levels (non-participation, occasional participation, and nearly/full attendance) coded as dummy variables; both the dependent and mediating variables were continuous. (5) Correlations between the independent and control variables were low (Spearman’s r < 0.5), and variance inflation factors (VIFs) were all less than 2, indicating that multicollinearity was minimal and unlikely to affect the predictive accuracy or explanatory power of the regression models. Statistical significance was set at *p* < 0.05.

### Data analysis

The dependent variable was continuous, and multiple linear regression models were used for analysis. Multiple linear regression is a statistical method primarily used to examine the relationships between a continuous dependent variable and its influencing factors (25). By estimating the regression coefficients of each independent variable, it is possible to quantify the predictive effect and direction of the independent variables on the dependent variable, thereby revealing the relative contribution of each factor. To examine whether physical exercise clock-in is associated with an increase in MVPA, the following model (Equation 1) was constructed for analysis.

$$\text{mvpa}=\alpha +\beta_{1}\text{attendance}+\beta_{2}\text{sportsactive}+\beta_{3}\text{gender}+\beta_{4}\text{residence}$$ (1)

In this analysis, *mvpa* serves as the dependent variable, indicating participants’ MVPA levels. *Attendance* denotes the physical exercise clock-in, while variables such as *sportsactive*, *gender*, and *residence* represent characteristics of personal demographics and other control variables that may influence MVPA. *α* denotes the intercept, and β *_1–4_* are the partial regression coefficients. The study employed a hierarchical regression approach to construct three regression models: Model 1 included only physical exercise clock-in without control variables; Model 2 added control variables for basic individual characteristics, based on Model 1; and Model 3 further incorporated the active sporting population variable. The results of the model analysis are presented in Table 3.

**Table 3:** Regression analysis results predicting MVPA levels among undergraduates.

Variable	Model 1			Model 2			Model 3		
	*b*	*SE*	*β*	*b*	*SE*	*β*	*b*	*SE*	*β*
Total sample (*n* = 468)									
*attendance (0)* Ref									
*attendance (1)*	4.868	6.729	0.045	5.031	6.234	0.046	2.748	6.477	0.025
*attendance (2)*	23.051^***^	6.146	0.233	21.621^***^	6.059	0.219	18.751^**^	5.938	0.190
Constant	43.513^***^	5.317		43.901^***^	6.114		40.640^***^	5.999	

*attendance (0)* represents the non-participation group, *attendance (1)* represents the occasional participation group, and *attendance(2)* represents the nearly full/full attendance group; ^***^p < 0.01, ^**^p < 0.05.

The regression results showed that, across different model specifications, the non-participation group was used as the reference category. In Model 1, which included only physical exercise clock-in, the results indicated that, compared with the non-participation group, students in the nearly full or full attendance group exhibited significantly higher MVPA levels (*b* = 23.051, 95% CI [10.97, 35.13], *p* < 0.01). In Model 2, after further controlling for basic individual characteristics, the positive effect of nearly full or full attendance on MVPA remained significant (*b* = 21.621, 95% CI [9.71, 33.53], *p* < 0.01). Model 3 additionally incorporated active sporting population as a control variable, and the results showed that nearly full or full attendance in physical exercise clock-in continued to significantly predict higher MVPA (*b* = 18.751, 95% CI [7.08, 30.42], *p* < 0.05). Although the regression coefficients decreased across models, the effect remained statistically significant. In contrast, the regression coefficients for the occasional participation group did not reach statistical significance in any of the three models.

Taken together, these findings suggest that, after controlling for basic individual characteristics and active sporting population, higher frequencies of exercise clock-in may be associated with higher MVPA levels among undergraduates, whereas low-frequency participation does not appear to show a significant association with MVPA.

### Robustness test

To address potential sample selection bias and test the robustness of the multivariate regression results, Propensity Score Matching (PSM) was employed as a supplementary analysis to examine the robust association between high-frequency exercise clock-in participation and MVPA. Specifically, three matching methods—nearest neighbor matching (k = 1), radius matching (caliper = 0.01), and kernel matching—which are widely applied in PSM (26, 27), were used to match samples from the treatment group (attendance2) and the control group (attendance0). The outcome variable was MVPA. Covariates included in the PSM were gender, residence location, and active sporting population.

As shown in Table 4, the estimated average treatment effects on the treated (ATT) were 19.48, 16.34, and 16.72, respectively, all statistically significant at the 5% level. Furthermore, the ATT estimates from the three methods were directionally consistent and numerically similar, with only minor variations reflecting differences in the matching algorithms. These findings indicate that high-frequency participation in physical exercise clock-in is associated with higher MVPA levels among undergraduates. Further calculation revealed that, compared to undergraduates in the control group, the MVPA levels of undergraduates in the treatment group increased by approximately 44.77, 32.56, and 33.48%, respectively. In summary, the estimates obtained via the PSM method are consistent with the conclusions from the multivariate regression analysis. By effectively mitigating sample selection bias, these results further strengthen the robustness of the study’s findings.

**Table 4:** ATT of PSM for the entire sample.

Methods	Samples	Treated	Controls	Diff.	S. E.	T-stat
Nearest neighbor matching	Unmatched	66.5639098	43.5129088	23.051001	6.62975148	3.48^***^
	Matched	62.9931153	43.5129088	19.4802065	6.93460834	2.81^***^
Radius matching	Unmatched	66.5639098	43.5129088	23.051001	6.62975148	3.48^***^
	Matched	66.5357143	50.1922224	16.3434919	6.74386688	2.42^**^
Kernel matching	Unmatched	66.5639098	43.5129088	23.051001	6.62975148	3.48^**^
	Matched	66.6916376	49.9679239	16.7237137	6.6979402	2.50^**^

The balance test (SMD < 0.1) and common support test were passed. ***p < 0.01, **p < 0.05.

The regression model indicates that high-frequency participation in exercise clock-in significantly predicts undergraduates’ MVPA. However, the underlying mechanism through which this relationship operates warrants further exploration. Within the analytical framework of Self-Determination Theory (SDT), it was proposed that physical exercise clock-in is associated with greater repetition and stability of exercise behaviors, thereby providing the necessary conditions for the development of exercise habits (7, 8). As individuals repeatedly engage in these activities and gradually experience positive outcomes, such as physical adaptation, emotional improvement, or enhanced competence, exercise behaviors initially driven by external constraints could be transformed into habitual behaviors with lower cognitive costs and greater automaticity (9, 28). This transformation may subsequently relate to higher MVPA levels. To examine this hypothesis, a mediation path model (Equations 2−4) was constructed in which exercise habits were included as a mediating variable. Nonparametric percentile bootstrapping (5,000 resamples) was conducted using the SPSS macro PROCESS (version 2.16) to enhance the robustness of the mediation effect estimates and to test the significance of the mediation effects.

$$\begin{array}{c} \;\;\\ \text{mvpa}=i_{1}+\beta_{1}\text{attendance}+\beta_{2}\text{gender}+\beta_{3}\text{residence}+e_{1}\\ \; \end{array}$$ (2)

$$\text{selfexercise}=i_{2}+\beta_{1}\text{attendance}+\beta_{2}\text{gender}+\beta_{3}\text{residence}+e_{2}$$ (3)

$$\begin{array}{c} \text{mvpa}=\begin{array}{c} i_{3}+\beta_{1}\text{attendance}+\beta_{2}\text{selfexcercise}+\\ \beta_{3}\text{gender}+\beta_{4}\text{residence}+e_{3} \end{array}\; \end{array}$$ (4)

*mvpa* represents participants’ MVPA levels and serves as the dependent variable. *Attendance* represents a physical exercise clock-in. *Selfexercise* represents exercise habits, quantified by the frequency of autonomous exercise. The variables *gender* and *residence* represent control variables related to personal characteristics. The terms *i_1-3_* correspond to the intercepts of each regression equation, while β *_1–4_* represent the partial regression coefficients for the respective variables, and *e_1-3_* denote the random error terms. The results of the model analysis are presented in Table 5.

**Table 5:** Mediating role of exercise habits between exercise clock-in and MVPA.

Mediation Path	Effect	95% CI	
		Lower	Upper
Total samples (*n* = 468)			
*attendance (0)* Ref			
*attendance (1)* → *selfexcercise* → *mvpa*	0.79	−2.1505	3.6496
*attendance (1)* → *mvpa*	4.24	−8.4428	16.9210
*attendance (2)* → *selfexcercise* → *mvpa*	5.99^a^	2.4983	10.4049
*attendance (2)* → *mvpa*	15.63	3.8006	27.4527
Females (*n* = 357)			
*attendance (0)* Ref			
*attendance (1)* → *selfexcercise* → *mvpa*	1.42	−1.1413	4.6122
*attendance (1)* → *mvpa*	3.09	−9.9446	16.1264
*attendance (2)* → *selfexcercise* → *mvpa*	5.61^a^	1.9598	10.443
*attendance (2)* → *mvpa*	16.10	3.8100	28.3928
Males (*n* = 111)			
*attendance (0)* Ref			
*attendance (1)* → *selfexcercise* → *mvpa*	1.10	−1.8643	4.0217
*attendance (1)* → *mvpa*	2.75	−10.2134	15.5982
*attendance (2)* → *selfexcercise* → *mvpa*	5.20^a^	1.6038	9.8125
*attendance (2)* → *mvpa*	14.80	2.517	26.8846

*attendance (0)* represents the non-participation group, *attendance (1)* represents the occasional participation group, and *attendance (2)* represents the nearly full/full attendance group; ^a^represents that the mediation effect is statistically significant.

As shown in Table 5, compared with the non-participation group, the mediating effect value of the nearly full or full attendance group on undergraduates’ MVPA levels through exercise habits was 5.99 (95% CI [2.50, 10.40]). The 95% bootstrap confidence interval excluded zero, indicating a significant mediation effect. Additionally, after the mediating variable was incorporated into the model, the direct effect on MVPA levels was 15.63 (95% CI [3.80, 27.45]), and its 95% bootstrap confidence interval also did not include zero. In contrast, for the occasional participation group, the mediation effect was not significant (95% CI [−2.15, 3.65]), and the direct effect also became nonsignificant after the mediator was included. Further subgroup analysis stratified by gender revealed a consistent pattern. In both males and females, high-frequency participation demonstrated a significant mediation effect through exercise habits, while the direct effect remained significant, indicating partial mediation. In contrast, occasional participation showed no significant direct or indirect effects in either subgroup.

These results suggest that when undergraduates reach a high level of participation, physical exercise clock-in may be associated with the development of exercise habits, which in turn could be related to higher MVPA levels. In contrast, low-frequency participation does not yield corresponding effects.

## Discussion

This study examined the associations between physical exercise clock-in and undergraduates’ daily MVPA as well as their physical exercise habits, based on self-report questionnaire data collected from a sample in Henan Province. Regression analyses revealed a positive dose–response relationship: greater participation frequency in the exercise clock-in was significantly associated with higher weekly MVPA accumulation (*b* = 18.751, 95% CI [7.08, 30.42], *p* < 0.05). Furthermore, path analysis revealed a significant indirect effect—exercise habits mediated the relationship between physical exercise clock-in and MVPA (indirect effect = 5.99, 95% CI [2.50, 10.40]). Further subgroup analysis stratified by gender revealed a consistent pattern. These results align with the theoretical premise that externally mandated accountability promotes adherence to regular exercise sessions and extends the duration of cumulative exercise, thereby increasing total energy expenditure and physiological load within fixed timeframes—ultimately elevating MVPA levels. Collectively, these findings suggest two distinct benefits associated with the clock-in program: (1) it may be linked to the development of sustained exercise habits through behavioral reinforcement, and (2) it may be associated with higher levels of objectively meaningful physical activity. Importantly, these findings are suggestive of a potential role for external regulatory mechanisms in cultivating long-term physical activity behaviors among adolescents and young adults, though further research is needed to establish causality.

In this study, high-frequency participation in physical exercise clock-in was significantly associated with a higher level of MVPA among undergraduates, whereas low-frequency participation was not significantly associated with MVPA. These findings suggest that the effectiveness of a clock-in physical exercise program may hinge on sustained, regular engagement rather than sporadic participation. High frequency participation appears to reflect a pattern of consistent exercise scheduling and satisfied cumulative weekly duration, leading to an elevated MVPA level with higher total energy expenditure. In contrast, infrequent or irregular participation is likely indicative of insufficient physical activity accumulation, resulting in failure to reach the threshold required for a measurable impact on the MVPA. Moreover, high-frequency exercise is related to progressive physiological adaptations, including improvements in cardiorespiratory fitness (29) and muscular endurance (30). These factors may help individuals to sustain higher intensities and longer durations in subsequent sessions, potentially contributing to greater overall physical activity engagement.

Indeed, regular physical activity not only enhances undergraduates’ physical fitness but also shapes their exercise-related behavioral patterns and attitudes toward physical activity. Empirical evidence has demonstrated that consistent participation in the mandatory exercise clock-in programs is significantly and positively associated with the development of habitual exercise behavior among undergraduates. Although it is plausible that students with pre-existing exercise habits are more likely to comply with the clock-in requirement, path analysis supports a theoretically consistent interpretation: sustained engagement is associated with patterns that resemble the internalization of exercise motivation—where externally imposed contingencies gradually become personally endorsed. This process is theoretically grounded in Organismic Integration Theory (OIT) (31), a sub-theory of SDT (8). OIT conceptualizes extrinsic motivation as existing along a continuum—from external regulation to integrated regulation—depending on the extent to which behavioral regulations are internalized and assimilated into one’s sense of identity and volition (32, 33). It should be noted, however, that our data did not directly measure motivational internalization; therefore, this interpretation remains theoretically plausible rather than empirically tested.

During early implementation, the clock-in requirement typically operates as external regulation: undergraduates comply primarily to avoid penalties, fulfill academic requirements, or meet institutional expectations. Under such conditions, behavior remains largely controlled, and perceived autonomy is comparatively low. Nevertheless, these structured external contingencies provide a stable behavioral scaffold and repeated opportunities for engagement. For individuals with initially low exercise motivation or high behavioral inertia, such requirements serve as an essential catalyst for initiating participation—establishing the foundational routine necessary for subsequent motivational internalization (34, 35). Moreover, as students persist in the program, they often experience positive feedback related to physical well-being, emotional satisfaction, or perceived competence; such experiences can facilitate the transformation of externally driven behavior into increasingly autonomous forms of self-regulation. Consequently, exercise clock-in programs may function not only as instruments of external control but also as facilitators of behavioral repetition and stabilization—thereby creating favorable conditions for habit formation and long-term increases in overall physical activity levels. Upon successful internalization, exercise behavior undergoes a qualitative shift: what once required conscious effort and external prompting evolves into a self-sustaining, automatic habit (28). Critically, such internally grounded habits extend beyond the temporal and institutional boundaries of the program itself—students with established routines not only attend scheduled sessions but also exhibit greater spontaneous physical activity across diverse daily contexts.

Notably, this study found no empirical evidence supporting the concern raised by certain critics—that mandatory exercise requirements undermine individuals’ attitudes toward physical activity (36–38). Nevertheless, although the observed positive association between exercise clock-in participation and habitual physical activity is statistically robust, it does not definitively preclude the possibility of adverse effects under specific contextual or individual circumstances. The absence of detectable negative outcomes may largely stem from the institutional framing of the exercise clock-in system as a behavioral scaffold rather than an end in itself. Its primary purpose is to provide undergraduates with a stable foundation for behavior initiation and repeated practice; its ultimate objective is to cultivate an autonomous, self-sustaining exercise habit that persists independently of external monitoring. Consequently, physical education instructors and relevant program coordinators must deliver progressive, developmentally appropriate guidance to support students’ transition from compliance-driven engagement to volitional, self-regulated behavior maintenance. Such scaffolding is essential to ensure that the clock-in system meaningfully contributes to the long-term development of sustainable physical activity patterns among undergraduates. Furthermore, institutional design intentionally incorporates bounded yet meaningful opportunities for student choice to attenuate the potentially controlling aspects of mandatory requirements. For example, while core activity standards remain unchanged, students may select preferred exercise modalities, timings, locations, or modes of completion. This calibrated autonomy supports fundamental psychological needs—particularly competence, relatedness, and especially autonomy—thereby strengthening behavioral identification and internalization (39, 40). However, this recommendation is derived from theoretical reasoning (SDT) and prior literature rather than direct empirical evidence from the present data. Experimental or quasi-experimental studies that systematically vary the degree of autonomy support are needed to determine whether such strategies enhance internalization and long-term physical activity maintenance beyond what is achieved by mandatory clock-in alone.

## Limitation

This research has several limitations. First, the sample data were collected from a single comprehensive university in Henan Province, where the clock-in program specifically involved campus running monitored via apps and contributed to course grades. This limits generalizability to programs with greater activity variety, to institutions where clock-in does not affect grades, and to non-Chinese cultural contexts with different attitudes toward mandatory exercise. Although our gender-stratified analyses showed consistent results, the sample was imbalanced; future studies should recruit larger and more balanced samples, particularly oversampling males, and test the findings across diverse program designs and cultural settings. Second, although PSM was applied to reduce potential sample selection bias and enhance the comparability between groups, the cross-sectional survey design still limits the ability to determine the temporal and causal relationships among the variables. Future research should employ longitudinal or follow-up designs to further verify the causal links between variables. Third, MVPA were primarily assessed through self-reports, which may introduce recall bias and social desirability effects, thereby compromising data accuracy. Subsequent research may integrate objective monitoring tools, including fitness trackers and smartwatches, to obtain more precise physical activity measurements. Fourth, approximately 7% of the samples were excluded because certain physical activity measurements fell significantly outside a reasonable range. Although this approach helped reduce bias introduced by extreme values, it also resulted in some data loss, which may have affected the generalizability of the findings. Finally, although a core dimension of exercise habit measurement is behavioral repetition (41), operationalizing habits solely by weekly frequency still neglects the crucial features of automaticity, contextual cueing, and reduced cognitive effort (42). Future research should employ validated habit strength measures (e.g., Self-Report Habit Index, SRHI) for better theoretical alignment.

## Conclusion

This study draws on survey data from 468 undergraduate students at a university in Henan Province, China, to examine the association among physical exercise clock-in, exercise habits and undergraduates’ MVPA levels, as well as the psychological mechanisms underlying this relationship. The results indicate that high-frequency participation in exercise clock-in is significantly and positively associated with higher MVPA levels, whereas low-frequency participation shows no statistically significant association. Furthermore, exercise habits partially mediates the link between high-frequency clock-in participation and MVPA. Further subgroup analysis stratified by gender revealed a consistent pattern. These findings suggest that the clock-in system may function not only as a compliance mechanism but also as a behavioral scaffold that supports the gradual internalization and stabilization of habitual physical activity. Collectively, the evidence offers preliminary support for the possibility that the program may help establish durable, self-sustaining exercise patterns among undergraduates, and might thereby contribute to long-term improvements in population-level physical activity. For future implementation, higher education institutions are advised to adopt a dual-focus design framework: upholding structural accountability while simultaneously cultivating autonomy-supportive conditions. Specifically, integrating meaningful choice (e.g., in exercise modality, timings, or mode of verification), embedding non-controlling positive reinforcement (e.g., personalized feedback, milestone recognition), and nurturing an inclusive, competence-affirming campus environment can facilitate undergraduates’ motivational progression—from external regulation toward identified and integrated regulation—ultimately promoting volitional, enduring engagement in physical activity.

## Data Availability

The raw data supporting the conclusions of this article will be made available by the authors, without undue reservation.
