# Supplementary material for: Do clock-in for physical exercise in campus help in building undergraduate exercise habits? Evidence from undergraduates in Henan, China
Source: Front Public Health. 2026 Jun 1;14:1844179. doi: 10.3389/fpubh.2026.1844179 (PMC13267197; doi:10.3389/fpubh.2026.1844179)
Supplement: Supplementary file 1 [file Supplementary_file_1.docx]

1. Gender

| ○male | ○female |
| --- | --- |

2. Residential location

| ○urban |
| --- |
| ○rural  3. During the past seven days, on how many days and for approximately how long each day did you engage in vigorous physical activity (e.g., running, heavy weight lifting)  (Please fill in the blanks)  4. During the past seven days, on how many days and for approximately how long each day did you engage in moderate physical activity (e.g., biking, brisk walking) |

(Please fill in the blanks)

5. How often have you participated in the university’s exercise clock-in program this semester?

○Never participated

○Occasionally participated

○Almost full attendance or full attendance

6. Apart from physical education classes and on-campus exercise clock-in, how many times per week do you engage in physical exercise?

○0

○1

○2

○3

○4

○5

○6

○7 times

7. Apart from physical education classes and on-campus exercise clock-in, how much time per week do you spend engaging in physical exercise?

○Not at all

○Less than 10 minutes

○11-20 minutes

○21-30 minutes

○31-45 minutes

○46-60 minutes

○61 minutes or more

8. Apart from physical education classes and on-campus exercise clock-in, how intense is your usual physical exercise?

○Not at all

○vigorous intensity (e.g., breathless and sweating)

○Moderate-to-vigorous intensity (e.g., slightly breathless and slightly sweating)

○Low intensity (e.g., no breathlessness or sweating)
